# Cross-cultural adaptation of the Italian version of the “Child and Youth Mental Health Instrument for Developmental Disabilities” (I-ChYMH-DD)

**DOI:** 10.1186/s13052-026-02224-7

**Published:** 2026-03-14

**Authors:** Elisabetta Sforza, Chiara Leoni, Elena De Rosa, Jacopo Bellani, Germana Viscogliosi, Antonella Di Paola, Marta Zampino, Rita Fischetto, Marika Pane, Donato Rigante, Serenella Servidei, Guido Primiano, Roberta Onesimo, Angelo Carfì, Zampino Giuseppe

**Affiliations:** 1 Department of Geriatrics, Centro Medicina dell’Invecchiamento, Fondazione Policlinico Universitario A. Gemelli IRCCS, 00168 Roma, Italy; 2 Department of Life Sciences and Public Health, Faculty of Medicine and Surgery, Università Cattolica del Sacro Cuore, 00168 Rome, Italy; 3 Pediatric Unit, Department of Woman and Child Health and Public Health, Fondazione Policlinico Universitario A. Gemelli IRCCS, 00168 Roma, Italy; 4 Center for Rare Diseases and Birth Defects, Department of Woman and Child Health and Public Health, Fondazione Policlinico Universitario A. Gemelli IRCCS, 00168 Roma, Italy; 5 National Institutes on Aging, National Institutes of Health, Baltimore, MD USA; 6 Istituto di Biologia e Genetica Generale, Medicina e Chirurgia, Università degli Studi di Bari “Aldo Moro”, Bari, Italy; 7 Centro Clinico Nemo Pediatrico, Fondazione Policlinico “A. Gemelli” IRCCS, Rome, Italy; 8 Institute of Neurology, Fondazione Policlinico Universitario A. Gemelli IRCCS, 00168 Rome, Italy

**Keywords:** Transition to adult care, Adolescents, Personalized medicine, Validation, Scale

## Abstract

**Background:**

The Child and Youth Mental Health and Developmental Disability ChYMH-DD is a psychometrically rigorous tool for care planning for children with developmental disabilities. It allows paediatricians and other healthcare professionals to perform a comprehensive assessment of children functional status and family burden.

**Aim:**

The aim of this study was to translate, culturally adapt, and preliminarily assess the Italian version of the ChYMH-DD for use in clinical settings involving pediatric patients with developmental disabilities.

**Methods:**

The original version of the ChYMH-DD was translated and cross-culturally adapted through five stages: (1) initial translation, (2) synthesis of the translations, (3) back translation, (4) expert committee, and (5) test of the pre-final version.

**Results:**

In the present study we successfully translated and adapted the ChYMH-DD into the Italian language. Translation of the questionnaire was performed without significant difficulties. During the third stage, no gross inconsistencies and conceptual errors as compared to the original version were highlighted.

**Conclusion:**

The Italian version of ChYMH-DD increase access to a valid and homogeneous assessment for children of Italian-speaking families and can support clinical decision-making. The Italian ChYMH-DD has potential to improve patients’ quality of life and quality of the care received for children with developmental disabilities.

## Introduction

Developmental disabilities (DDs) are severe chronic mental or physical impairments that begin before age 22 years, and are likely to continue throughout life [1]. The range of deficits varies from very specific learning limitations to a global impairment of social skills and intelligence [2] and frequently occur concurrently [3]. For example, individuals with an autism spectrum disorder (ASD) frequently face cognitive impairments, while many children with attention-deficit/hyperactivity disorder also have specific learning disorders [4].

DDs encompass both neurodevelopmental disorders (NDDs), including intellectual disability (ID), and other early-onset conditions not classified as NDDs, including cerebral palsy, epilepsy, congenital sensory impairments, and genetic syndromes such as Down syndrome and Fragile X syndrome [5].

With reference to the Italian context, the recent survey conducted by Bacherini and colleagues indicates that the number of children reporting DDs is rising among school children [6]. Specifically, ID is the most common type of cognitive limitation in elementary and middle school, affecting 42% of students receiving special education. In Western countries ID affects 1.5 to 2% of the population and represents an important health burden [7]. Individuals with DDs report poorer health and are more vulnerable to mental illness [8] although they encounter numerous barriers to accessing health promotion and disease prevention programs [9].

As often DDs results in functional limitations in three or more major life activity areas, namely those activities that are necessary for basic functional activity of daily living (ADLs) [10], there is a need for a combination of special, interdisciplinary, or generic care services as well as individualized support plans. DDs require a comprehensive assessment oriented towards assistance focusing on specific needs, which calls for a standardized method that serves this purpose [11].

The Child and Youth Mental Health and Developmental Disability (ChYMH-DD) is a comprehensive, clinician-rated, standardized and multidisciplinary mental health measure for children and youth, ages 4–18 years: it is a standardized assessment tool intended to support comprehensive care planning, outcome measurements, quality indicators [12]. To date, the ChYMH-DD still needs to be validated for the Italian-speaking population. Herein, the purpose of our study was to translate and culturally adapt the ChYMH-DD within an Italian population of pediatric patients with DDs.

## Methods

The study was conducted in accordance with the Declaration of Helsinki and approved by the local ethics committee within the framework of the “AD MAIORA” project [PNRR-MR1-2022-12376346]. The STrengthening the Reporting of OBservational studies in Epidemiology guidelines (STROBE) were used in the preparation of this study.

### Description of the ChYMH-DD

The ChYMH-DD includes items related to the needs, areas of risk, functioning, and strengths of children and youth: it is part of an integrated health information system in which psychometrically sound scales and algorithms are embedded within the instrument to support clinical decision-making. The items are tailored to the needs of children and youth with DDs and mental health concerns, both in inpatient and outpatient settings, as part of the standard of care. In keeping with other interRAI instruments [12], items employ specific observation periods in order to provide reliable and valid measures of clinical characteristics that reflect the child’s strengths, preferences, and needs. Importantly, some items address the recency and frequency of symptoms prior to and within the last 3 days. Whitin the questionnaire, there is also an option to indicate that symptoms are present but not exhibited within the last 3 days. Responses for items are constrained, almost always binary or ordinal. The ChYMH-DD also includes the interRAI Adolescent Supplement Assessment Form and item-by-item guide for its use. This supplement to the ChYMH-DD core assessment includes items used to collect information about the following domains: Substance Use or Excessive Behavior; Parental Status (Youth as Parent); Independence in Daily Activities; Prevention, Service Utilization, Treatments; Strengths; Mental Health and Well-Being [12].

### Translation and cross-cultural adaptation of the ChYMH-DD

The translation of the questionnaire and its cross-cultural adaptation was carried out according to the Guidelines for the Process of Cross-Cultural Adaptation of Self-Report Measures [13, 14]. The process consisted of five different stages: initial translation (stage 1); synthesis of the translations (stage 2); reverse translation (stage 3); expert committee (stage 4); and test of the prefinal version (stage 5).

#### Stage 1-Initial translation

After obtaining permission from the authors of the original version, the ChYMH-DD was first translated from English into Italian by both a bilingual psychologist experienced in developmental disabilities and a bilingual translator with no medical or clinical background. The two professionals produced two independent translations of the tool.

#### Stage 2-Synthesis of the translations

The translations were compared and, after addressing any disagreement, a consensus for one common translation was reached. Informal or local terms or complex unfamiliar terminologies were avoided.

#### Stage 3-Back-translation

A consistent translation was ensured by the results obtained through the backtranslation, performed by two distinct English native translators who were unaware of the original scale. Specifically, the common Italian translation achieved in the previous phase was translated back into English to ensure that the translated version was reflecting the same item content as the original versions.

#### Stage 4-Expert committee

Subsequently, a multidisciplinary panel met to produce the prefinal Italian version of the ChYMH-DD (I- ChYMH-DD). The panel, composed of clinicians and translators, reconciled any existing differences between translations.

#### *Stage 5-Test of the prefinal version (face validity*)

After the completion of the fourth stage, the readability of the tool was checked between experienced clinicians (namely, Italian-speaking clinicians with research and scientific publications in the area of paediatrics or intellectual disabilities) working at the Rare Disease Unit of the Department of Life Sciences and Public Health in the Policlinico Universitario Agostino Gemelli IRCCS. The consent form was sent by email and all those who accepted to participate in the study received the tool in an electronic form.

The equivalence analysis by experienced clinicians occurred both quantitatively, comparing the English version with the Italian and qualitatively, through comments and suggestions for each item [15].

## Results

The translation of the questionnaire advanced without significant difficulties. During the third stage, no gross inconsistencies and conceptual errors with the original version were highlighted. During the fourth stage, the professionals discussed and evaluated each item from their viewpoints. The wording of Italian questions was specifically chosen to minimize any influence to the participants’ responses, but relevant and applicable in the Italian linguistic context. In addition, to address potential cultural incongruities and ensure conceptual equivalence, the expert committee reached consensus on translation choices that preserved both semantic accuracy and cultural appropriateness. In terms of content, some items were modified due to their Country-specific nature. Specifically, in section A-Identification Information, item n. 5 “Agency Identifier” was considered not applicable and removable thus not affecting the final score. In the same section, answers to item n. 6 “Current Payment Sources” were adapted to the Italian context, leaving two answer options: (a) “national health service” or option (b) “other”. Answer “Child protection agency” to question n. 10 was retained not applicable in the Italian context as well as the question n. 3 “Aboriginal Identity” in section B “Intake and Initial History”. In the same section, three possible answer options were identified for question n. 4 “Primary Language”, namely ‘Italian’, ‘other’ and ‘Italian sign language’. In section B, for question n. 14 “Admitted From and Usual Residence”, answer n.2 “Board and care” was deleted as retained equivalent to answer n. 9 “Residential care facility” within the Italian context. In section J “Communication”, we changed the example given to answer n. 2d “Any adapted or manually coded language” from “Signed Exact English” to “Italiano Segnato Esatto”. Answer “Special education class” to question n. 2 in Section S “Education”, was retained not applicable in the Italian context. A panel of seventeen experts—including paediatricians, child neuropsychiatrists, psychologists, geriatricians, speech therapists, and geneticists—evaluated the semantic and idiomatic equivalence of the translated instrument and judged it adequate, confirming that the translation preserved the meaning of the original expressions. The majority also agreed that the situations described in the items were consistent with those typically encountered in the Italian cultural context, thereby supporting cultural relevance. Conceptual equivalence was likewise deemed satisfactory, with the content of the items considered easily understandable. The expert panel further assessed the instrument section by section using a 4-point Likert scale (1 = not clear, 2 = somewhat clear, 3 = fairly clear, 4 = very clear). Overall, 91.6% of the ratings were ≥ 3 (fairly clear or very clear), with 56.6% rated as very clear.

## Discussion

Children and young people with DDs are a large and growing population. According to the 2023 report published by the World Health Organization (WHO) and UNICEF, there are approximately 317 million children and adolescents with health conditions contributing to developmental disabilities globally [16]. Individuals with DDs experience poorer health outcomes, have unmet healthcare needs due to fragmented health care systems and experience vast disparities in terms of health services [16].

A comprehensive approach to care planning that incorporates standardized instruments such as the ChYMH-DD aligns with international strategies to optimize the health and wellbeing of children with developmental disabilities. The ChYMH-DD is a clinician-rated assessment for individuals aged 4–18 years, developed within the InterRAI network—a collaboration of more than 100 researchers, clinicians, and policy experts from over 35 countries—designed to support care planning, monitor outcomes, and inform quality indicators [12]. The instrument has demonstrated robust psychometric properties, with inter-item reliability coefficients (Cronbach’s α) of ≥ 0.70 across all scales and strong criterion validity, evidenced by moderate-to-strong correlations (*r* ≈ 0.60) with established measures of emotional and behavioral functioning, including the Child Behavior Checklist (CBCL; anxious/depressed and social problems scales), the Brief Child and Family Phone Interview (BCFPI; managing mood and self-harm scale), and the Social Skills Improvement System (SSIS; internalizing, externalizing, and self-control subscales) [17–18]. In the present study we successfully translated and adapted the ChYMH-DD into the Italian language.

Of importance, the ChYMH-DD belongs to a wider and integrated set of instruments that share a common assessment language, conceptual basis, clinical emphasis, data collection approach, data elements, and care planning protocols. This enables the assessment of homogenous groups of patients across their lifespan [12]. Specifically, it makes possible to follow the patient with DDs in the transition phase from childhood to adulthood and obtain a reliable set of information that can be used individually for case documentation and to inform the transition program planning [19–22]. From 1978 National Health System (NHS) primary care pediatricians in Italy take care of infants and adolescents from birth 16 years of age [23]. Crucial in the transition from child to adult health care providers is to identify health and developmental needs and ensure preventive care supports consequently [24].

Data generated can be used also for research purposes, given the relative lack of studies examining the well-being of people with DDs transitioning to adulthood [8, 25, 26]. Moreover, longitudinal data on transition generated by the consistent use ChYMH-DD may be aggregated at the population level to benchmark performance for internal quality improvement, accreditation and public reporting [27–32].

As reported by Stewart and colleagues, key applications of ChYMH-DD include a case mix classification [12]. Considering this last element, a case mix classification allows to capture the effect of DDs on the Italian National Health Service (SSN) and to estimate the relative resource allocation [33–35]. To date, case-mix classification data allows to predict more accurately health service utilization and costs also thanks to the development of machine learning approaches [36–38].

In view of prevention and promotion of well-being, Stewart and colleagues administered the ChYMH-DD to comprehensively assess early signs of mental health impairments in children with and without DDs and to develop an algorithm to identify factors associated with distress among caregivers of children and youth referred for in the Canadian mental health services [39].

This study has limitations. Although we applied rigorous cross-cultural translation procedures, we did not validate the Italian ChYMH-DD in a clinical sample. Thus, while semantic, conceptual, and cultural equivalence with the original instrument was ensured, the reliability and criterion validity of the translated version remain unconfirmed. In addition, face-validity testing involved only clinicians from a single institution, limiting generalizability. Further studies in diverse clinical settings with systematic psychometric testing are needed to confirm the reliability, validity, and applicability of the Italian ChYMH-DD.

## Conclusion

This study translated and cross-culturally adapted the Child and Youth Mental Health and Developmental Disability (ChYMH-DD) assessment into Italian using a rigorous multi-phase process to ensure semantic, conceptual, and cultural equivalence. The resulting tool appears appropriate for use in clinical settings with children and adolescents with developmental disabilities, providing a standardized framework to guide individualized assessment, care planning, and family engagement. Furthermore, the Italian ChYMH-DD may facilitate more robust data collection to inform service planning, case-mix classification, and the development of structured transition pathways to adulthood, ultimately supporting efforts to improve healthcare access, quality, and coordination for this population.

## Data Availability

Data are available from the corresponding author upon reasonable request.

## Abbreviations

ASD: Autism spectrum disorder
ChYMH-DD: The child and youth mental health and developmental disability
DDs: Developmental disabilities
I-ChYMH-DD: Italian version of the “child and youth mental health instrument for developmental disabilities”
ID: Including intellectual disability
NDDs: Neurodevelopmental disorders
